# Sustainable Design of Urban Rooftop Food-Energy-Land Nexus

**DOI:** 10.1016/j.isci.2020.101743

**Published:** 2020-10-27

**Authors:** Rui Jing, Astley Hastings, Miao Guo

**Affiliations:** 1 Department of Chemical Engineering, Imperial College London, London, SW7 2AZ, UK; 2 Key Lab of Urban Environment and Health, Institute of Urban Environment, Chinese Academy of Sciences, Xiamen, 361021, China; 3 Institute of Biological and Environmental Sciences, University of Aberdeen, Aberdeen, AB24 3FX, UK; 4 Department of Engineering, Strand Campus, King's College London, London, WC2R 2LS, UK

**Keywords:** Environmental Science, Environmental Technology, Environmental Assessment, Economics

## Abstract

Urban rooftop functional design offers a promising option to enable multi-function urban land-use to deliver multiple ecosystem services, e.g., food production by rooftop agriculture and energy supply by installing photovoltaic (PV) panels. To identify the best rooftop utilization strategy considering multiple decision criteria and understand the impact of rooftop solution on the design of urban energy systems, we propose a whole system modeling framework that integrates biogeochemical simulation and multi-objective energy system optimization. We apply the framework to evaluate three rooftop agriculture options, namely, basic rooftop farming, unconditioned greenhouse, and conditioned greenhouse, and one rooftop energy supply option, i.e., PV panels, for an urban energy eco-design case in Shanghai, China. Enabling rooftop agriculture options brings more flexibility to the design and operation of energy systems. PV panels provide cost-optimal solutions, whereas conditioned greenhouse potentially delivers environmentally sustainable land-use by contributing to climate regulation ecosystem services.

## Highlights

• Propose a holistic approach for optimizing urban rooftop multi-functional land-use
• Biogeochemical simulations are integrated with energy system design optimization
• Rooftop agriculture options can increase the flexibility of energy system design
• PV is a cost-optimal option, and conditioned greenhouse offers a GHG-optimal option

## Graphical Abstract

**Figure:**
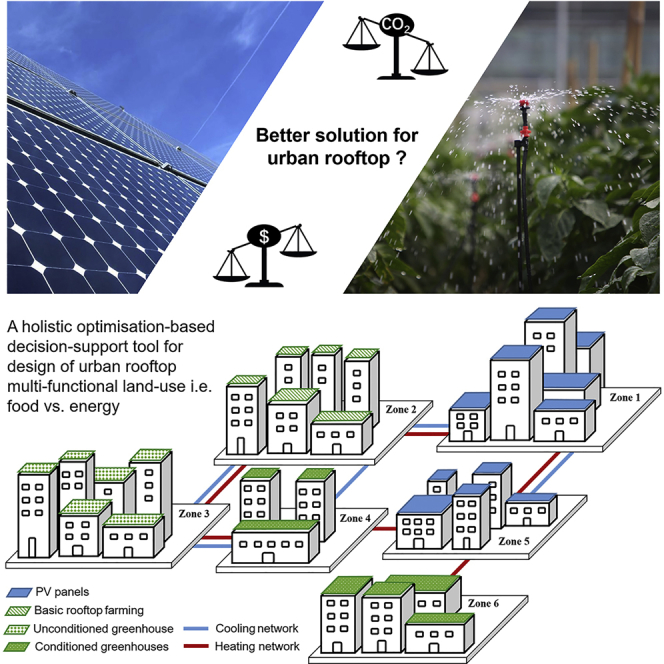

## Introduction

Landscapes generate multiple benefits for human society and individual well-being including housing, transportation, and a wide range of ecosystem services (ES) (Mace et al., 2012; MillenniumEcosystemAssessment, 2005). These services can be broadly categorized into four categories, i.e., provisioning services, e.g., food and energy; regulating and supporting services, e.g., climate and water regulation and waste recycling; and cultural services, e.g., recreational value. Although the need to incorporate such ES into decision support at different spatial scales is increasingly recognized, their value is often overlooked in real-world land-use planning applications (Bateman et al., 2013). Over the last decade, urban ES and urban agriculture have received increasing attentions as two-thirds of the overall population is expected to be urbanized in 2050 (Cortinovis and Geneletti, 2018; Hansen et al., 2019; IEA, 2019). With rapid urbanization and the projected 50% increase of population in the twenty-first century globally (UN, 2017), food and energy demand are expected to increase 50% and 30%, respectively, between now and 2050 (EIA, 2019; Grafton et al., 2015). This will increase resource supply stress and affect land scarcity and natural ecosystems (Folberth et al., 2020). A transformation from traditional farming toward sustainable land management and urban agriculture systems is necessary where multi-functional land-use systems and urban food production sites enable sustainable food supply for the urban consumption centers (Orsini et al., 2014). However, such transformation is hindered by conflicting ES, such as climate regulation versus the food and energy provision, which compete on the limited urban land resources (Acuto et al., 2018). How this urban populace will be sustainably fed and energized is a vital focus for governments, urban planners, and academia.

Urban rooftops offer alternative resources for multi-functional land-use (e.g., housing and urban agriculture), but its potential benefits have not been well explored. Two promising solutions are proposed in this study—(1) implementing rooftop agriculture for food production and (2) installing photovoltaic (PV) panels for energy supply. Implementing rooftop agriculture has the potential to bring a range of benefits such as reducing urban heat island effect (Coccolo et al., 2018; Hossain et al., 2019), modulating microclimates (Duarte et al., 2015; Mauree et al., 2019), and mitigating atmospheric greenhouse gas emissions (GHG, e.g., CO_2_) (Sanyé-Mengual et al., 2015). It can also strengthen social connections between neighborhoods (Benis et al., 2018), as well as of people with nature (Mincyte and Dobernig, 2016). The installation of PV panels can generate power and change the role of buildings from energy consumers to prosumers (Poruschi and Ambrey, 2019). In the meantime, such distributed power generation could enhance the energy security (Kakran and Chanana, 2018; Zeng et al., 2019) and increase public awareness of climate change (Benis et al., 2017). A synergetic integration of rooftop agriculture and PV offers a solution to couple land-competing food energy with other ES (e.g., carbon and water recycling) into urban landscape decision-making by optimizing multi-functional land-use.

Despite the potential multiple benefits from integrative rooftop agriculture-PV system, sustainably planning rooftop space to meet land-competing food-energy demands in the urban context requires a whole systems approach; however, the research on rooftop system design integrated with urban food-energy-land nexus remains largely unexplored. Recent research advances include the life cycle assessment of different rooftop utilization options (Corcelli et al., 2019; Goldstein et al., 2016; Sanjuan-Delmás et al., 2018), agent-based simulation and environmental evaluation of individual options (Mittal et al., 2019; Yeo and Lee, 2018; Zeng et al., 2008), and optimization-simulation integrative modeling of agricultural land-use and ES with conflicting decision criteria, e.g., economic and environmental objectives (Garcia et al., 2019; Groot et al., 2018; Kaim et al., 2018). More recent research efforts have been made to co-locate renewables (solar, wind) with agricultural production in drylands (Barron-Gafford et al., 2019; Ravi et al., 2016) and propose holistic approaches to assess energy-food-land nexus at a larger scale (Liu et al., 2018; Van Vuuren et al., 2019). Nevertheless, research on the integrative urban rooftop system in the literature is sparse. Several modeling gaps emerged from a review of state-of-the-art literature: (1) synergetic integration of rooftop agriculture and energy to meet urban land-competing ES; (2) system implications of different rooftop design options, e.g., impacts of design option on urban energy system; and (3) systems approach with multiple design criteria to inform decision-making on urban rooftop design at community or district scales. This calls for an integrated rooftop design underpinned by a whole systems approach, which considers the multi-functional land-use and demands for multiple ES (e.g., food and energy provisioning services and carbon regulation services) (Edmondson et al., 2020; Howells and Rogner, 2014; Kinnunen et al., 2020).

**Figure 1:**
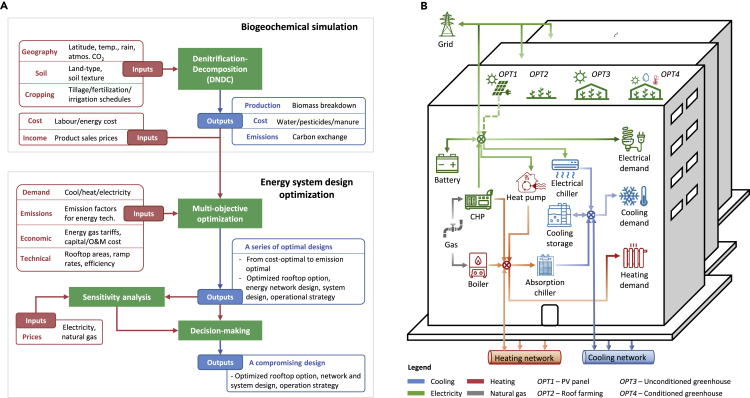
Overview of Integrated Modeling Framework and Illustration of the Energy Hub with Four Available Rooftop Options (A) The proposed tool consists of two modules, i.e., biogeochemical module and energy system module. The biogeochemical module simulates the yields, inputs, and emissions of three rooftop agriculture options. This information is fed to the energy system module, by which the optimal results including the best rooftop option, energy network design, energy system configuration, and system operation strategy can be obtained. (B) The electricity, cooling, and heating demands of all buildings within one zone are served by an energy hub. The energy hub model is generalized, including six commonly used energy supply technologies, two energy storage technologies, the interactions to the grid, as well as energy network availability (Jing et al., 2019a). On the rooftop of each building, four options are available assuming the bearing capacity of rooftop is sufficient. The optimization results will determine the best choice of the rooftop option as well as the optimal energy system configurations and hourly operational strategies.

We aim to address the abovementioned research gaps and present a multi-objective optimization framework to evaluate different rooftop utilization options considering the limited rooftop area. As shown in Figure 1A, a cross-disciplinary approach has been adopted in this study to bring mathematical optimization into urban planning decision-making and highlight the integration of denitrification-decomposition (DNDC) biogeochemical modeling with energy system optimization to inform rooftop planning. Specifically, we use a biogeochemical simulation to project the crop yields, inputs, and emissions of different rooftop agriculture options and further feed this information into the system design optimization model. The design optimization model optimizes the rooftop option, energy network design, energy system configuration, and operation strategy. The optimal designs are further validated by sensitivity analysis considering energy price volatility. Different objective functions lead to a set of optimal designs, and one trade-off optimal design is selected by the decision-making procedure (more details in Transparent Methods).

Figure 1B illustrates the neighborhood-level energy system with four available rooftop options. The rooftop options are closely related to the energy system design. Installing PV panels (OPT1) generates green electricity to reduce the reliance on grid electricity supply, whereas implementing rooftop agriculture options (OPT2∼OPT4) has the potential to deliver economic benefits, mitigate carbon emissions, and reduce buildings energy demands. Hence, both rooftop energy and agriculture options have the potential to contribute positively to the energy system in terms of economic and environmental footprints. A case study in Shanghai, China, demonstrates the applicability of the framework and provides insights into the optimal rooftop utilization to deliver multiple ES (i.e., food and energy provisioning). In the Results section, we have briefly described the case specifications and then analyzed the biogeochemical simulation results. This is followed by the energy system optimization modeling and detailed analyses of the optimal energy system design. We have then analyzed the impacts of different rooftop options on energy system design and presented sensitivity analyses results. The Results section is followed by Discussion, Conclusion, and Limitations of the Study sections. Related modeling details are presented in the Transparent Methods section.

## Results

### Case Study Specifications

**Figure 2:**
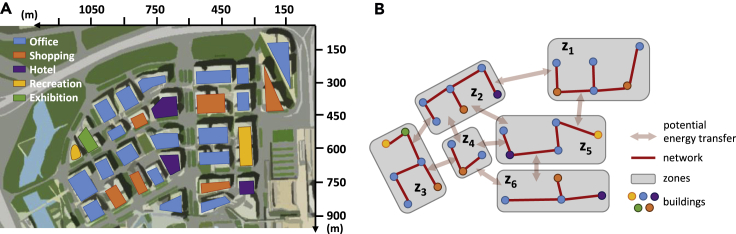
Basic Information of the Urban Neighborhood, Nodes (i.e., Buildings), and Network (A) Five categories of buildings, as well as the corresponding locations and available rooftops, are plotted in Figure 2A. The available rooftop areas of each building vary between 900 and 2,500 m^2^ based on the measurement from design documents as reported in Jing et al. (2019c). (B) Each node represents one building. All nodes are classified into six clusters (i.e., zones) by k-means technique according to the locations of each building (Jing et al., 2019c).

An urban neighborhood with 30 large commercial buildings in Shanghai, China, (Figure 2A) is used as a case study to demonstrate the model functionality, where the project lifespan is assumed as 20 years. Time-of-use electricity tariff and gas price (see Supplemental Information Figure S3) are obtained from representative data of the local market (Zhang et al., 2019). Each year is divided into three periods, namely, summer, winter, and transition period. Cooling is supplied in summer, heating is supplied in winter, and no heating or cooling demand is there in the transition period (see Supplemental Information Figure S5). A typical day is modeled for each period, and it is equally split into hourly intervals with varying solar conditions (see Supplemental Information Figure S4). All buildings are clustered into six zones (Figure 2B); the urban energy system needs to be designed optimally to simultaneously fulfill the energy demands of all zones including electricity, cooling, and heating. Within each zone, one energy hub, located at the node with the largest energy demand, can be installed to supply the energy demand of that zone via an optimally designed energy network. Energy can be transmitted between energy hubs if necessary. The shortest length of the network and guaranteed connection of all buildings is achieved in each zone by the minimum spanning tree technique (Unternährer et al., 2017). All energy hubs are connected to the utility grids as well. Four options are available for designing rooftop utilization strategies in each zone.

**Figure 3:**
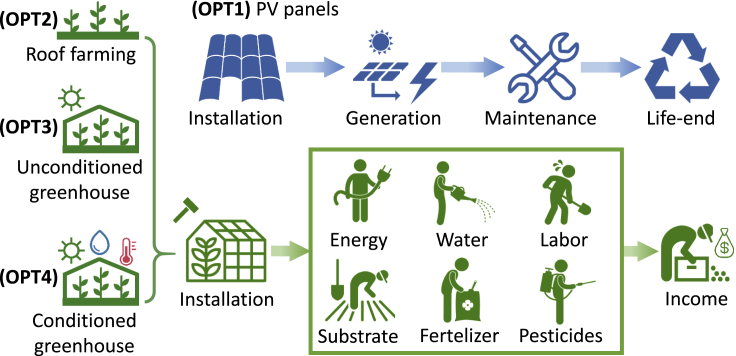
Definitions of Rooftop PV and Agriculture Options For all three agriculture options, we consider the life cycle cost including installation, various processes of planting, as well as the tomato yields income. The yield, operational cost, and emission saving potential are calculated from the biogeochemical simulation. For PV option, we consider the life cycle cost, and the profit comes from generating onsite electricity. The emission saving can be achieved by using sustainable electricity instead of the grid electricity.

Figure 3 visualizes the definitions of four available rooftop options, which are closely related to the energy hub design. Installing PV panels (OPT1) can generate green electricity to reduce the reliance on grid electrical energy. In contrast, implementing rooftop agriculture options (OPT2∼OPT4) is expected to bring additional economic benefits, mitigate carbon emissions, and reduce buildings' cooling and heating demands. Hence, both rooftop energy and agriculture options have the potential to contribute positively to energy hubs in terms of economic and environmental footprints.

All energy hubs are allowed to make different choices among the four available rooftop options in our modeling framework. These four available rooftop options, i.e., PV panels and rooftop tomato farming, are further defined in Table 1 and visualized in Figure 3.

**Table 1:** Definition of Available Rooftop Options

Index	Option	Note
OPT1	PV panels	Install PV panels and generate sustainable electricity
OPT2	Basic rooftop farming of tomato	Basic agriculture cultivation system configuration without greenhouse or optimal control of temperature, lighting, and humidity
OPT3	Unconditioned greenhouse of tomato	Steel greenhouse structures with a plastic film cover but without temperature, lighting, and humidity control
OPT4	Conditioned greenhouse of tomato	Advanced greenhouse systems with well-configured temperature, lighting, and humidity control

### Biogeochemical Simulation Results

We model the tomato cultivation on rooftops considering that China is one of the main tomato producers and consumers worldwide, and that tomato is rich in nutrition and acts as a key resource of daily vitamin C intake (Wikipedia, 2020). The biomass yields and C partitioning between seed, stem, leaves, and roots obtained from the DNDC model (Li et al., 1992) simulations for one crop cycle (approximately 150 days) are given in Table 2 based on daily temperature and rainfall conditions (see Supplemental Information Figure S1).

**Table 2:** DNDC Simulated Biomass Yields for Different Rooftop Agriculture Options

Index	Option	NEE (kg C/ha/year)	Biomass Yield (kg C/ha/year)			
			Seed	Stem	Leaves	Roots
OPT-2	Basic rooftop farming	−405	170	104	104	95
OPT-3	Unconditioned greenhouse	−974	405	248	248	225
OPT-4	Conditioned greenhouse	−1,841	745	455	455	414

DNDC simulated the daily carbon fluxes (see Supplemental InformationFigure S2). Gross primary production (GPP) represents the total amount of carbon fixed by photosynthesis (Wang et al., 2012), whereas the net ecosystem exchange (NEE) of carbon is equivalent to the difference between GPP and ecosystem respiration (ER) (Deng et al., 2014; Elsgaard et al., 2012). ER is the biotic conversion of organic carbon to carbon dioxide by all organisms within the ecosystem (Yvon-Durocher et al., 2012), accounting for the plant respiration by root, shoot, and leaf as well as the microbial heterotrophic respiration. In DNDC, the plant respiration is simulated by a daily time step considering the effects of environmental drivers such as the atmospheric temperature and nitrogen availability. Meanwhile, the microbial heterotrophic respiration is calculated by simulating soil organic carbon decomposition (Deng et al., 2014). As shown in Table 2, DNDC projected negative NEE for one crop cycle, which indicated a net uptake of CO_2_ by the plant-soil ecosystem. The NEE values vary with the options—roof farming, unconditioned greenhouse, and conditioned greenhouse can achieve −405, −974, and −1,841 kg C/ha/year, respectively. These simulation results demonstrate the beneficial effects of elevated CO_2_ level in OPT4 on net carbon sequestration by plant-soil ecosystem. By incorporating the DNDC simulation results into the multi-objective optimization, the modeling framework enables urban rooftop utilization solutions to account for the biogeochemical carbon cycling.

### Multi-objective Energy System Optimal Design

**Figure 4:**
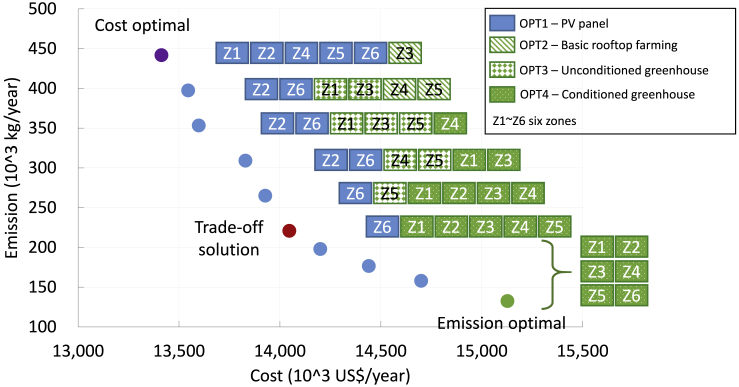
Pareto Frontier of Different Optimal Solutions These solutions denote the optimal design of the whole energy system fulfilling the electricity cooling and heating demands of 30 buildings. The cost-optimal (cost-minimization), emission-optimal (CO_2_-minimization), and a trade-off solution are marked by different colors.

By integrating DNDC simulation outputs into multi-objective optimization and techno-economic parameterization, a series of different system design and rooftop utilization strategies are derived. As plotted in Figure 4, a Pareto frontier represents the trade-off between cost optimal and GHG minimization objectives, where the system design and selection of rooftop options vary significantly. Note that the cost includes the annual operation expense and the capital expenses amortized over the assumed lifetime of the project, i.e., 20 years. To further enable the decision-makers to articulate their preference of multiple decision criteria and lead to optimal solution to address the trade-offs, we apply the Technique for Order Preference by Similarity to an Ideal Solution (TOPSIS) method to choose one trade-off solution with maximum rationality of selection (Jing et al., 2019b) (more details in Transparent Methods).

Objective function moves from cost-optimal to GHG minimization, whereas the rooftop utilization transits gradually from an OPT1-PV panel-dominated solution to OPT1∼OPT4 hybrid solutions and finally selects an OPT4-conditioned greenhouse solution. Considering economic feasibility, rooftop PV panel (OPT1) is the preferable solution for most of the zones with one zone exception where roof farming (OPT2) is selected. Along the Pareto frontier, basic rooftop farming (OPT2) and unconditioned greenhouse solution (OPT3) are gradually selected by most zones as the environmentally favorable choice when considering the trade-offs between economic and GHG objectives. At the emission minimization mode, all zones selected the conditioned greenhouse (OPT4) as their rooftop solution. The underlying reasons are that OPT1-PV panel tends to bring more economic benefit to the whole system compared with the other rooftop agriculture options (OPT2∼OPT4), whereas OPT2∼OPT4 could lead to more GHG reductions. Among agriculture options, OPT2-basic rooftop farming offers economically competitive option (close to OPT1-PV panel). OPT4 delivers superior GHG reduction effects despite the relatively high costs.

In the meantime, combined rooftop agriculture options (OPT2∼OPT4) could significantly increase the flexibility of energy system design. As shown in Figure 4, when only OPT4 is selected, the system performance varies within a relatively narrow range (14,200–15,200×10^3^ USD/year for cost and 135–200 × 10^3^ kg/year carbon emission) by just changing the energy system design. While different rooftop agriculture (OPT2∼OPT4) and energy options (OPT1) are available, the system design flexibility significantly improved with the cost and GHG objectives varying within a wider range of 13,400–15,200×10^3^ USD/year and 135–445 × 10^3^ kg/year, respectively.

### Impact of Rooftop Options on Energy System Design

**Figure 5:**
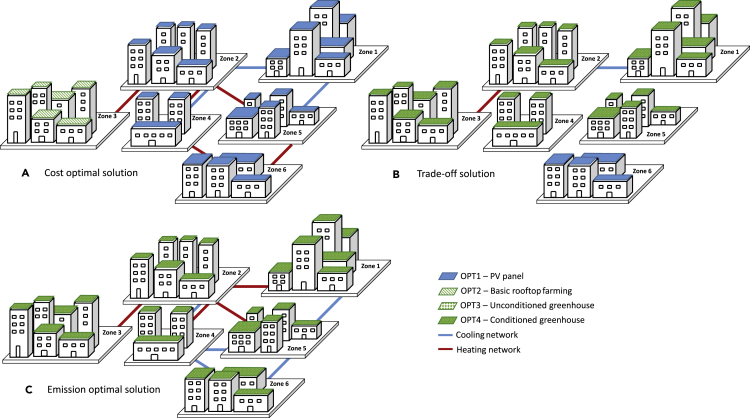
Optimal urban rooftop solution and corresponding energy network design with different objectives (A–C) (A) Cost-optimal solution, (B) trade-off solution, and (C) emission-optimal solution.

**Figure 6:**
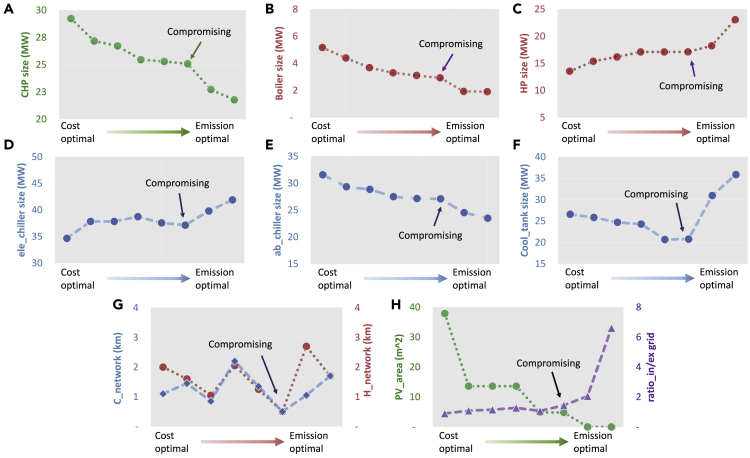
Size of the Installed Technologies for Different Solutions along the Pareto Frontier (A–H) (A) CHP, (B) boiler, (C) heat pump, (D) electric chiller, (E) absorption chiller, (F) cooling storage tank, (G) cooling and heating network length, and (H) PV panel area and grid electricity import/export ratio.

The rooftop options, as well as heating and cooling network design, for three representative solutions are visualized in Figure 5, which is consistent with the observation in Figure 4. No obvious trends can be concluded in terms of cooling and heating network design from three representative optimal solutions on the Pareto frontier. A further investigation for system implications of rooftop solutions on the energy system configuration and network design is illustrated in Figure 6.

In Figure 6, the technology sizing choices are expressed as a function of the objectives switching from cost-optimal to CO_2_ emission-optimal. Several observations are summarized below.

1. With the objective moving from cost-optimal to GHG-optimal, the installed capacity of combined heating and power (CHP) and boiler dropped gradually as shown in Figures 6A and 6B. This can be partially explained by the natural gas-based technologies (CHP and boiler) offering cost-competitive design options but embedding higher CO_2_ emissions. To achieve lower emission designs gradually, the installed capacities of CHP and boiler have to decrease. Consequently, less residual heat is available for absorption chiller to generate cooling; a reduced installation capacity was also observed in Figure 6E. Meanwhile, PV panel (OPT1) gives its place to agriculture options (OPT2∼OPT4) to fulfill the gradually higher requirement of the GHG objective.
2. As less capacity of the absorption chiller is installed, a larger capacity of the electric chiller is required to fulfill the cooling demand. With the increase in electric chiller capacity, more electricity is required. Due to the lower capacity of CHP, more electricity has to be imported from the grid with a higher import/export ratio as shown in Figure 6H.
3. The size of the cooling storage tank decreases followed by a rapid increase at the compromising point. Meanwhile, no obvious trends are found in cooling and heating network design; the cooling storage tank seems not to play signification role in the network design.
4. The variation degree for the capacity of each technology is different before and after the compromising point. From cost-optimal to trade-off solution, selecting different rooftop solutions (OPT1∼OPT4) produces more impacts on system design than the energy technologies themselves. By selecting different agriculture solutions (OPT2∼OPT4), CO_2_ emission can be reduced efficiently. Consequently, the change of installed capacities for energy technologies is relatively moderate. Once the optimal solution passes the compromising point, the rooftop solution is constant (all select OPT4–conditioned greenhouse), whereas capacities of energy technologies vary to further reduce CO_2_ emissions.

Overall, constrained by limited land resources particularly in urban areas, rooftop offers alternative options for multi-functional land-use. As presented in this study, by integrating agriculture and energy systems with urban rooftops, the land not only delivers housing benefits for human society but also has the potential to bring multiple ES (energy and food provisioning and climate regulation ES). Different rooftop options (OPT1∼OPT4) impact the ES benefits (income and climate regulation) significantly. Energy provisioning option (OPT1) brings the highest income achieving the cost-optimal rooftop land-use solution. Agriculture options (OPT2∼OPT4) bring more climate regulation ES benefits than OPT1, among which OPT4 with elevated CO_2_ concentration is shown as a favorable choice to achieve a GHG minimization solution.

### Sensitivity Analysis

**Figure 7:**
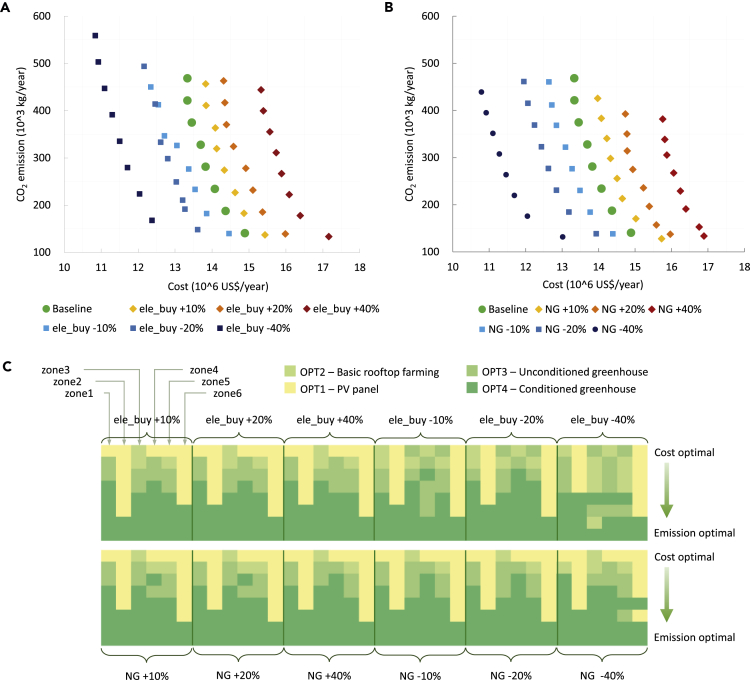
Electricity and Natural Gas Prices' Sensitivities on System Cost and Emissions Assume the grid electricity price (ele_buy) and the natural gas price (NG) vary between −40% and +40% from the baseline. For both prices, six scenarios, i.e., −40%, −20%, −10%, +10%, +20%, and +40%, are evaluated. (A) A series of Pareto frontiers led by different electricity prices. (B) A series of Pareto frontiers led by different natural gas prices. (C) Rooftop solutions' variations for different price-varying scenarios.

Sensitivity analyses were carried out to understand the system implications of the electricity and natural gas prices. As demonstrated in Figures 7A and 7B, the prices of the grid electricity and the natural gas significantly impact the trade-offs between cost and emissions performances. Generally, with the increase in energy price (electricity and natural gas), the whole system costs increase. Despite the variation in prices, all scenarios achieved similar GHG performances. For each scenario, moving toward the minimal cost, the emission level increases with a reduction in energy prices. This can be explained by the higher quantity of grid electricity purchased, which offers cost-efficient energy but induces higher GHG emissions compared with onsite power generation.

Figure 7C illustrates rooftop solutions along the Pareto frontier for each scenario. Although the system performances vary with energy prices, the rooftop use strategies remain relatively stable. This can explain the Pareto frontiers in Figures 7A and 7B, which are relatively evenly distributed. Overall, sensitivity analysis suggests a constant optimal solution for rooftop utilization, regardless of the energy prices variation, where PV panels (OPT1) are selected as a cost-optimal option and conditioned greenhouse (OPT4) is selected by all rooftop design to achieve minimized GHG scores.

## Discussion

To achieve food-energy-land nexus sustainability in an urban context, rooftop agriculture and energy systems offer promising solutions through multi-functional land-use design strategies. Four design options have been explored in the current study including solar PV panels for power generation (OPT1) and rooftop agriculture systems without and with controlled greenhouse (OPT2∼OPT4). A cross-disciplinary approach has been applied to integrate biogeochemical simulation and mathematical optimization into urban energy planning decision-making framework. The developed Mixed Integer Linear Programming model enables simultaneous optimization of rooftop utilization strategies and the whole energy system design to assess the design trade-off between the minimized costs and GHGs. This essentially represents a trade-off between provisioning and regulatory ES. Our research highlights that the PV panel (OPT1) and the rooftop greenhouse with controlled CO_2_ concentration, temperature, lighting, and humidity (OPT4) offer an economically competitive and environmentally sustainable choice, respectively.

The system configurations and dispatch strategies differ significantly with the consideration of multiple conflicting objectives. Our results agree with the findings from previous studies on design of urban energy systems considering energy technologies only (Jing et al., 2019b; Li et al., 2018; Terlouw et al., 2019). Moreover, we find that enabling rooftop agriculture systems offers more flexibility for energy system design when economic and GHG objectives are considered. By merely selecting different rooftop options with a minor capacity variation of energy technologies, the GHG emission of the whole system can be reduced efficiently; once the rooftop solution is constant, the capacities of energy technologies need to vary significantly to further achieve lower emission design. Besides, earlier study has found that food production could be more beneficial than energy generation in Mediterranean climates through cost-benefit analysis of rooftop solutions only (Benis et al., 2018); however, the impact of rooftop solutions on the whole urban energy system has not been considered. The results could be case specific depending on various conditions, e.g., climate, type of buildings, food and energy prices, etc. All above observations, in turn, highlight the importance of developing tools that can bring land-competing systems (e.g., food and energy) and conflicting objectives into a whole system decision support framework to inform urban landscape design.

Notably, only carbon sequestration by the rooftop agricultural systems is accounted for in the model, where attributional carbon counting approach has been followed. However, the plants, e.g., tomato cultivated in rooftop agriculture systems can avoid the arable land-use elsewhere, which further leads to avoidance of GHG emissions caused by land-use. Thus, following a consequential carbon counting approach, the saving effects caused by land-use GHG avoidance could enhance the environmental competitiveness of rooftop agriculture systems. This study only accounts for the GHG emissions of the operation phase, which often dominates life cycle GHGs of an energy system (Wang et al., 2015). Future efforts are needed to integrate comprehensive life cycle assessment and multiple environmental impact indicators into the modeling framework.

## Conclusion

Overall, the proposed modeling framework integrates for the first time biogeochemical simulation and multi-objective optimization to understand the implications of environmental variables (e.g., temperature, atmospheric CO_2_ concentration) and crop-environment interaction on urban energy systems design. In this study, we first modeled different tomato cultivation options using biogeochemical simulation and developed a neighborhood-level energy system optimization model; the biogeochemical simulation results were fed into energy system model to resolve the bi-objective optimization to address the trade-offs between cost optimal and GHG emission minimization. An illustrative case study demonstrates the applicability of the proposed decision-support tool and generates insights into the optimal design options for rooftop at a given urban neighborhood in Shanghai, China. Our research suggests that the multi-functional rooftop design from whole systems perspective enables urban food-energy-land sustainability to deliver food and energy provisioning, carbon regulation ES. The integration of agriculture options brings more flexibility to urban energy systems design when multiple conflicting objectives are considered. The PV panels provide cost-optimal rooftop solutions, whereas conditioned greenhouse potentially delivers environmentally sustainable land-use by contributing to climate regulation ES.

### Limitations of the Study

This study presented a modeling framework underpinned by biogeochemical simulation and energy systems optimization to inform the rooftop utilization options. Several emerging research directions are worth further investigation efforts and highlighted below:

(1) In the current study, all building rooftops were suitable for implementing rooftop farming and PV options from building structure perspective. These building rooftops were assumed to be exposed to the sun, whereas building heights and the possible shadow effects of adjacent taller buildings were not considered. However, such effects could play significant roles in some locations, and thus are worth exploring.
(2) The urban energy model we developed is based on a green-field case when designing a new building and energy system. However, an interesting research direction would be to compare new building and building retrofit. The process of retrofitting would involve a balancing of different design elements and their effects on the overall performance (e.g., energy demand, safety) of a building; thus the design criteria, design space for building retrofit, could be significantly different from rooftop design with new building. Despite the current research on a case study in the context of China, the modeling framework developed in this study could be applied to rooftop design and case studies in other urban or hinterland areas where the building patterns and underlying parameters and design criteria would vary with the region- or country-specific climate and geographical features.
(3) The optimization modeling framework developed in this study addresses the trade-off between economic and environmental objectives and considers food/energy provisioning and climate regulation ES. However, to enable such modeling framework to provide evidences for specific rooftop design solutions, inputs and feedback from multiple decision-makers (e.g., policy makers, urban planners, households) on model feasible spaces and decision criteria are important. This can be achieved by engaging multiple model users in interactive solution-searching settings to support informed decision-making. User interaction can be explored by developing a human-in-the-loop approach in multi-level modeling research to articulate the dynamic preferences of multiple decision-makers based on their gradually built understanding of the model topology and enable the solution search to be progressively directed toward the regions of interest.
(4) The current study integrated energy and agriculture system with urban rooftops to deliver housing benefits and multiple ES including energy and food provisioning and climate regulation ES. However, other potential design criteria, e.g., building safety, stability, and wider ES, e.g., water cycle-related ES, can be incorporated into the modeling framework proposed in this study and further developed in future research
(5) The current study focuses on PV and rooftop farming; however, other potential rooftop utilization strategies including renewable energy solutions, e.g., PV-wind hybrid system and recreation park, could be explored in future research investigations. The modeling framework developed in this study could be further expanded to include wider renewable energy systems in the energy system design optimization module and simulate other plant species in the biogeochemical simulation module.
(6) Another interesting future research direction is to consider urban microclimate conditions and its interaction with rooftop farming and energy systems. Either PV or vegetation on rooftops could affect the urban microclimate and consequently affect the energy performance of the urban neighborhood. For example, both rooftop PV and vegetation contribute to mitigation of the urban heat island effects and further lead to lower cooling demands as well as higher power output from PV (Berardi and Graham, 2020; Dong et al., 2020). Relevant research could be further embedded in both energy supply and demand-side of the proposed modeling framework to achieve a more holistic research.

### Resource Availability

#### Lead Contact

Further information and requests for resources and reagents should be directed to and will be fulfilled by the Lead Contact, Miao Guo (miao.guo@kcl.ac.uk).

#### Materials Availability

This study did not generate new unique reagents.

#### Data and Code Availability

The input data are available in Supplemental Information, and the code of energy system model associated with the article is available from the Lead Contact on reasonable request.

## Methods

All methods can be found in the accompanying Transparent Methods supplemental file.

## Supplemental Information

Document S1. Transparent Methods, Figures S1–S5, and Tables S1–S3

## Footnotes

Supplemental Information can be found online at https://doi.org/10.1016/j.isci.2020.101743.
