# Supplementary material for: Sustainable Design of Urban Rooftop Food-Energy-Land Nexus
Source: iScience. 2020 Oct 27;23(11):101743. doi: 10.1016/j.isci.2020.101743 (PMC7663218; doi:10.1016/j.isci.2020.101743)
Supplement: Document S1. Transparent Methods, Figures S1–S5, and Tables S1–S3 [file mmc1.pdf]

iScience, Volume *23*

Supplemental Information

Sustainable Design of Urban Rooftop Food-Energy-Land Nexus

Rui Jing, Astley Hastings, and Miao Guo

Transparent Methods

This Transparent Methods includes (1) setup and parameterisation of biogeochemical simulation, and (2) assumptions and formulations the energy system optimization model.

Biogeochemical simulation

Process-based biogeochemical model Denitrification-Decomposition tool (DNDC) is adopted in this study to simulate the plant growth and carbon and nitrogen cycles in response to environmental variables and management strategies (e.g. elevated atmospheric CO_2 concentration). The DNDC model is one of the most well-developed process-oriented biogeochemistry models and commonly utilized worldwide (Abdalla et al., 2009; Babu et al., 2006; Beheydt et al., 2007; Brown et al., 2002; Butterbach-Bahl et al., 2004; Butterbach-Bahl et al., 2001; Cai et al., 2003; Grant et al., 2004; Smith et al., 2002; Wang et al., 1997).

The DNDC model was first proposed by Li et al. (Li et al., 1992); over two-decade development, numerous changes have been implemented to DNDC model to bridge functional gaps and be adapted to region- or user-group specific versions UK-DNDC (Brown et al., 2002), DNDC-Europe (Guo et al., 2015) forest-DNDC, manure-DNDC (Li et al., 2012). A schematic family tree has been reviewed and presented by Gilhespy et al. (Gilhespy et al., 2014). A relatively complete suite of biogeochemical processes (e.g., plant growth, organic matter decomposition, fermentation, ammonia volatilization, nitrification, denitrification) has been embedded in the model, enabling computation of transport and transformations in plant-soil ecosystems. By linking with chemical engineering process design and life cycle assessment, DNDC has been applied to simulate biomass growth and carbon/nitrogen cycling in agro-ecosystems and their implications on bioproduct system sustainability (Guo et al., 2012; Guo et al., 2015). Within DNDC, the soil temperature, moisture and redox potential profiles driven by daily weather data are simulated by the soil climate sub-model considering the soil texture and plant's water demand. The crop growth and development driven by air temperature, soil water, and nitrogen supplement is simulated by the plant growth sub-model at a daily timestep. In the meantime, the decomposition sub-model tracks turnover of soil organic matters that produce CO_2 emitted from the soil as well as inorganic nitrogen released from mineralization. The other three sub-models calculate trace gas emissions from nitrification, denitrification, and fermentation, respectively. All six sub-models interact with each other to simulate the targeted ecosystem's water, C and N cycles. Overall, DNDC can predict the impacts of climate change or management alternatives on the soil biogeochemistry and the crop yield.

We use DNDC to simulate the daily tomato growth and daily net ecosystem exchange (NEE) of carbon based on the detailed parameterization and simulation setup as shown in **Table S1** in **Supplemental Information (SI)**. The 5-year (2011~2015) daily meteorological data (temperature, precipitation) for DNDC simulations were estimated based on the data obtained from the China meteorological data sharing service system (CMDC, 2018) and presented in **Fig. S1**, where the daily max-min temperature varies within a range of -5~37 °C and daily rainfall ranges between 0 and 25 cm.

The DNDC simulated daily NEE fluxes for one crop cycle (approximately 150 days) is presented in **Fig. S2** for different rooftop agriculture options. The annual yield and NEE (see **Table 2**) along with capital and operation cost breakdown are presented in **Table S2**. The derived income of tomato yield (calculated based on Eq. 10), the NEE, the capital and operation cost, are used to parameterize the energy system design optimization model.

Energy system design optimization

This study aims to optimize urban rooftop utilization to achieve multi-functional rooftop area use to deliver multiple ecosystem services, i.e. food and energy provisioning and climate regulation. To achieve this, we developed a multi-objective (i.e., cost and emission minimization) optimization model, which is a Mixed-Integer Linear Programming model and follows a bottom-up approach that optimizes the system design and operational strategy simultaneously. The optimization problem can be stated as follows:

Given a neighborhood of 30 buildings with known rooftop areas, locations, energy demands, weather conditions and available technology options for energy and food crop production, to ***determine*** the rooftop utilization, energy system design and operational strategy, to ***achieve*** a series of optimal system designs representing the trade-offs between two conflicting objectives i.e. the minimized annualized cost and the minimized carbon emissions. The overall electricity, cooling and heating demands of all buildings over an hourly interval time horizon must be fulfilled simultaneously, which bound the energy system design and the operational strategy. The rooftop areas and weather condition regulate the crop yields, CO_2 emissions and operational costs. The defined optimization problems investigate the cost-efficient and environmentally sustainable design and operational strategy considering capital inputs and operational configurations (e.g. energy transmission loss) and policy intervention (energy supply tariff).

Specifically, several features of the model are described below:

Energy Hub Mode. The model is developed following the energy hub mode, where 30 buildings are clustered into 6 zones by spatial clustering technique (i.e., K-means) based on the relative distances between buildings (Unternährer et al., 2017). Buildings are clustered to specific number of zones; the sum of distances between cluster centroid and buildings in that zone is minimal. Within each zone, an energy hub is assumed located at the building with the largest energy demands. The energy hub fulfils the energy demands of all buildings in that zone through an internal energy network (Perera et al., 2020), which is pre-optimized by Minimum Spanning Tree algorithm (Jing et al., 2019c). Meanwhile, each energy hub is flexible to connect to neighboring hubs if needed, depending on the optimization results. Hence, we essentially optimize the design, operation and interactions of 6 networked energy hubs along with the rooftop utilization solutions in the urban neighborhood.

Multi-objective optimization. The ε-constraint approach is applied to solve the bi-objective (i.e., cost and emissions) minimization problem. As derived in Eq. (1), the ε-constraint approach maintains the $f_1(x)$ as objective function, and converts the $f_2(x)$ to a constraint by introducing a parameter of ε. Hence, the bi-objective problem is converted to a typical single-objective problem [Jing et al., 2019a], $f_1(x)$ and $f_2(x)$ denote objective function of AC and ACE.

$$\begin{aligned} &\min \ f_1(x) \\ &\text{S.T.} \ \ f_2(x) \le \varepsilon \\ &\quad \text{and other constraints from original model} \end{aligned} \tag{1}$$

By minimizing $f_1(x)$ and $f_2(x)$ individually, the minimum and maximum values of $f_2(x)$ can be obtained as $f_2^{min}(x)$ and $f_2^{max}(x)$. Then, for each point N+1, the value of ε can be calculated by $\varepsilon = f_2^{max}(x) - \frac{f_2^{max}(x) - f_2^{min}(x)}{N}\mu$, where N is the number of self-defined intervals between minimum and maximum values of $f_2(x)$, μ=0,…,N.

Decision-making. The TOPSIS decision-making method is adopted to find one trade-off solution from a set of solutions on the Pareto frontier (Jing et al., 2019b). All solutions (i.e., points) are numbered by m in a n dimensional coordinate system in the first place. These points are then normalized and the TOPSIS method defines an ideal-point and a non-ideal point based on the distribution of the points. We further calculate the Euclidian distance (ED) of each point on the Pareto frontier to the ideal-point (ED_{m+}) and the non-ideal point (ED_{m-}), respectively, by Eq. 2(a-b). The point with relative farthest distance from the non-ideal point (i.e., biggest Y_m value) is selected as the trade-off solution (Eq. 2(c)) (Jing et al., 2018).

$$ED_{m+} = \sqrt{\sum_{n=1} (f_{m,n}^{\text{norm}} - f_n^{\text{ideal}})^2} \tag{2a}$$

$$ED_{m-} = \sqrt{\sum_{n=1} (f_{m,n}^{\text{norm}} - f_n^{\text{nadir}})^2} \tag{2b}$$

$$Y_m = \frac{ED_{m-}}{ED_{m-} + ED_{m+}} \tag{2c}$$

Sensitivity Analysis. Sensitivity analyses were conducted to validate the modelling outputs solutions considering the price variability of grid electricity and natural gas (Mavromatidis et al., 2018; Yue et al., 2018). Considering the computational time, an energy price variation range of -40% to +40% was assumed for both electricity and natural gas with an interval of every 10% fluctuation. Our optimization results suggest that the rooftop utilisation strategy is not sensitive to the energy price parameters; regardless of energy price variation, the PV panel and the conditioned greenhouse were modelled as cost-effective and GHG optimal solutions respectively.

Model Description. In the optimization model, energy demands and prices for each year, and energy conversion efficiencies (e.g. CHP efficiency) were assumed as constant. The DNDC-simulated crop yields were annualized, and the constant operational costs were assumed for plantation management (e.g. fertilization, irrigation). The key decision variables and parameters are defined in the **Table 3~Table 7** whereas the detailed parameterization is given in **Table S3**.

Table 3 Definitions of indices in the optimization model

Indices	Definitions
s	Sets of three representative seasons
h	Sets of 24 hours
i	Sets of zones
j	Sets of zones, $j \neq i$
t	Sets of energy supply devices, including PV, CHP, boiler (b), electric chiller (ec), absorption chiller (ac), heat pump (hp), battery storage (b-st), cooling storage tank (c-st)
k	Sets of three rooftop agriculture options (k=1 roof farming, k=2 unconditioned greenhouse, k=3 conditioned greenhouse)
$\overline{(\cdot)}$	Upper bound of variables

Table 4 Definitions of parameters in the optimization model

**Parameters**	**Definitions**
C^{CAP}	Unit capital cost [\$/kW] of CHP ($C_{\mathrm{CHP}}^{\mathrm{CAP}}$), boiler ($C_{\mathrm{b}}^{\mathrm{CAP}}$), electric chiller ($C_{\mathrm{ec}}^{\mathrm{CAP}}$),absorption chiller ($C_{\mathrm{ac}}^{\mathrm{CAP}}$), heat pump ($C_{\mathrm{hp}}^{\mathrm{CAP}}$), PV panel ($C_{\mathrm{pv}}^{\mathrm{CAP}}$) , heating and cooling network ($C_{\mathrm{pipe}}^{\mathrm{CAP}}$), battery storage ($C_{\mathrm{b\text{-}st}}^{\mathrm{CAP}}$), cooling storage tank ($C_{\mathrm{c\text{-}st}}^{\mathrm{CAP}}$); and unit capital cost [\$/m^2] of rooftop agriculture option (C_{k}^{CAP})
$DX_{i,j}$	Distance between zones
η	Efficiency of CHP (η^{CHP}), boiler (η^{b}), electric chiller (η^{ec}), absorption chiller (η^{ac}), heat pump (η^{hp}), PV panel ($\eta_{s,h}^{\mathrm{pv}}$), storage self-discharge ($\eta^{\mathrm{in\text{-}st}}$), storage charge/discharge ($\eta^{\mathrm{cha/disc}}$)
H-to-P	Heat-to-power rate of CHP
C_{h}^{NG}	Unit cost of natural gas [\$/kWh] at hour h for CHP ($C_{h}^{\mathrm{CHP\text{-}NG}}$), boiler ($C_{h}^{\mathrm{b\text{-}NG}}$)
C^{maint}	Maintenance cost [\$/kWh] of CHP ($C_{\mathrm{CHP}}^{\mathrm{maint}}$), boiler ($C_{\mathrm{b}}^{\mathrm{maint}}$), electric chiller ($C_{\mathrm{ec}}^{\mathrm{maint}}$), absorption chiller ($C_{\mathrm{ac}}^{\mathrm{maint}}$), heat pump ($C_{\mathrm{hp}}^{\mathrm{maint}}$), PV panel ($C_{\mathrm{pv}}^{\mathrm{maint}}$), battery storage ($C_{\mathrm{b\text{-}st}}^{\mathrm{maint}}$),cooling storage tank ($C_{\mathrm{c\text{-}st}}^{\mathrm{maint}}$)
CRF	Capital recovery factor for 15, 25, 30 years
C_{h}^{im}	unit price of grid electricity purchasing at hour h [\$/kWh]
C_{h}^{ex}	tariff for electricity sold back to grid at hour h [\$/kWh]
Ψ	Emission factor [kg/kWh] of the grid electricity (Ψ_{grid}) and natural gas (Ψ_{NG}); emission factor of k rooftop agriculture option (ψ_{k}^{agri})
A_i	Available roof area in i zones [m^2]
income$_{i,k}$	annual unit income for k rooftop agriculture option in zone i [\$/m^2/year]
opex$_{i,k}$	annual unit planting cost for k rooftop agriculture option in zone i [\$/m^2/year]
$\mathrm{Q}_{i,s,h}^{\mathrm{dem}}$	Demand in zone i at season s and hour h for heating ($\mathrm{Q}_{i,s,h}^{\mathrm{h\text{-}dem}}$), cooling ($\mathrm{Q}_{i,s,h}^{\mathrm{c\text{-}dem}}$)
$\mathrm{Q}_{i,s,h,k}^{\mathrm{roof}}$	Demand saved in zone i by k rooftop agriculture option for heating ($\mathrm{Q}_{i,s,h,k}^{\mathrm{h\text{-}roof}}$), cooling ($\mathrm{Q}_{i,s,h,k}^{\mathrm{c\text{-}roof}}$)
Lo^{pipe}	Thermal loss rate for cooling network ($Lo^{\mathrm{c\text{-}pipe}}$), heating network ($Lo^{\mathrm{h\text{-}pipe}}$)
$SRI_{s,h}$	Solar Radiation index at season s and hour h

M	The “big M” big enough values for M_1 and M_2

Table 5 Definitions of free variables in the optimization model

Variables	Definitions
AC	The objective of annualized cost
ACE	The objective of annualized greenhouse gas emissions

Table 6 Definitions of binary variables in the optimization model

Binary Variables	Definitions
$\varphi_{i,k}^{\text{agri}}$	=1 if select the *k* rooftop option in zone *i*
φ_{i}^{PV}	=1 if select the rooftop PV option in zone *i*
$\beta_{i,s,h}^{\text{CHP}}$	=1 if CHP is on for in zone *i* at season *s* hour *h*
$\chi_{i,s,h}^{\text{CHP}}$	=1 if CHP is switching from off to on in zone *i* at season *s* hour *h*
$\alpha_{i,s,h}^{\text{cha}}$	=1 if energy is charged into storage in zone *i* at season *s* hour *h*
$\alpha_{i,s,h}^{\text{disc}}$	=1 if energy is discharged from storage in zone *i* at season *s* hour *h*
$\delta_{i,s,h}^{\text{ex}}$	=1 if electricity is sold back to the grid in zone *i* at season *s* hour *h*
$\delta_{i,s,h}^{\text{im}}$	=1 if electricity is bought from the grid in zone *i* at season *s* hour *h*
$\delta_{i,j}^{\text{DH}}$	=1 if district heating network is built among zone *i* and *j*
$\delta_{i,j}^{\text{DC}}$	=1 if district cooling network is built among zone *i* and *j*
$\gamma_{i,s,h}^{\text{DH}}$	=1 if zone *i* is receiving heating via heating network at season *s* hour *h*
$\gamma_{i,s,h}^{\text{DC}}$	=1 if zone i is receiving cooling via cooling network at season *s* hour *h*

Table 7 Definitions of positive variables in the optimization model

Positive Variables	Definitions
CAPEX	The capital cost of the whole system
FC	The fuel cost
MC	The maintenance cost
GC	The grid electricity cost
FI	The food yield income
$CAP_{i,t}$	The installed capacity of energy technology *t* in zone *i*
$E_{i,s,h}^{\text{pv}}$	The electricity output from PV panel in zone *i* at season *s* hour *h*
$E_{i,s,h}^{\text{CHP}}$	The electricity output from CHP in zone *i* at season *s* hour *h*
$Q_{i,s,h}^{\text{hp}}$	The heating output from heating pump in zone *i* at season *s* hour *h*
$Q_{i,s,h}^{\text{b-heat}}$	The heating output from boiler in zone *i* at season *s* hour *h*

$Q_{i,s,h}^{\text{ac-cool}}$	The cooling output from absorption chiller in zone *i* at season *s* hour *h*
$Q_{i,s,h}^{\text{ec-cool}}$	The cooling output from electric chiller in zone *i* at season *s* hour *h*
$Q_{i,s,h}^{\text{in-st}}$	The cooling stored in storage tank in zone *i* at season *s* hour *h*
$E_{i,s,h}^{\text{in-st}}$	The electricity stored in battery in zone *i* at season *s* hour *h*
$E_{i,s,h}^{\text{im}}$	The electricity bought from the grid in zone *i* at season *s* hour *h*
$E_{i,s,h}^{\text{ex}}$	The electricity sold back to the grid in zone *i* at season *s* hour *h*
$Q_{i,j,s,h}^{\text{hf}(i,j)}$	The heating flow from zone *i* to *j* at season *s* hour *h*
$Q_{j,i,s,h}^{\text{hf}(j,i)}$	The heating flow from zone *j* to *i* at season *s* hour *h*
$Q_{i,j,s,h}^{\text{cf}(i,j)}$	The cooling flow from zone *i* to *j* at season *s* hour *h*
$Q_{j,i,s,h}^{\text{cf}(j,i)}$	The cooling flow from zone *j* to *i* at season *s* hour *h*
$Q_{i,s,h}^{\text{re-heat}}$	The heating output from CHP in zone *i* at season *s* hour *h*
$Q_{i,s,h}^{\text{cha}}$	The cooling charge into cooling storage in zone *i* at season *s* hour *h*
$Q_{i,s,h}^{\text{disc}}$	The cooling energy discharged in zone *i* at season *s* hour *h*
$E_{i,s,h}^{\text{st-in}}$	The electricity charge into battery storage in zone *i* at season *s* hour *h*
$E_{i,s,h}^{\text{st-out}}$	The electricity discharged in zone *i* at season *s* hour *h*
$NG_{i,s,h}^{\text{b}}$	The natural gas consumed by boiler in zone *i* at season *s* hour *h*
$NG_{i,s,h}^{\text{CHP}}$	The natural gas consumed by CHP in zone *i* at season *s* hour *h*

Objectives. This study considers conflicting objectives, i.e., annualized cost and GHG emissions. The annualized cost (*AC*) is calculated as Eq. (3).

$$AC = CAPEX + FC + MC + GC - FI \tag{3}$$

where *CAPEX* represents capital cost, *FC* denotes fuel cost, *MC* is maintenance cost, *GC* is the grid cost, and *FI* defines food income.

The *CAPEX* includes the capital cost of all energy device, energy network, and the potential construction of different rooftop agriculture options as shown in Eq. (4). Assuming the interest rate of 6%, the *CAPEX* is further annualized by multiplying a capital recovery factor (*CRF*) as shown in Eq. (5). The service life of energy supply devices is assumed as 20 years, 15 years for rooftop agriculture, and 30 years for the energy network considering the corresponding durability. Eq. (6) ensures at most one rooftop option selected for each zone.

$$\begin{aligned} CAPEX = & \sum_i \sum_t CAP_{i,t} \times C_t^{\text{CAP}} \times CRF_t + \sum_i DX_{i,j} \times C_{\text{pipe}}^{\text{CAP}} \times CRF_{\text{pipe}} \\ & + \sum_{k=1,2,3} \sum_i \varphi_{i,k}^{\text{agri}} \times C_k^{\text{CAP}} \times CRF_{\text{agri}} \quad \forall t, i, j \neq i \end{aligned} \tag{4}$$

$$CRF = \frac{r \times (1+r)^n}{(1+r)^n - 1} \tag{5}$$

$$\sum_{k=1}^{3} \varphi_{i,k}^{\text{agri}} + \varphi_i^{\text{PV}} \le 1 \quad \forall i \tag{6}$$

where *i, j, t* denote zone number (*i* and *j*), and energy technologies (*t*), respectively. *k*=1~3 denotes three rooftop agriculture options. *CAP* indicates the installed capacity. DX represents the distance between zones. φ is a binary variable determining whether implementing one certain rooftop agriculture options or PV installations or not.

Fuel cost (*FC*) is determined by the gas consumption by all devices as derived from Eq. (7). Boiler and CHP consume gas, and the gas price for CHP is lower than boiler due to the policy incentive schemes in China favoring the distributed energy technologies (i.e., CHP).

$$FC = \sum_i \sum_s \sum_h [(\frac{E_{i,s,h}^{\text{CHP}}}{\eta^{\text{CHP}}} \times C_h^{\text{CHP-NG}} + \frac{Q_{i,s,h}^{\text{b-heat}}}{\eta^{\text{b}}}) \times C_h^{\text{b-NG}}] \quad \forall s, h, i \tag{7}$$

where *i s, h* denote the zones, seasons, and hours, respectively. E^{CHP} is the power generation from CHP, $Q^{\text{b-heat}}$ indicates the heating supply by boiler, η is the efficiency, and C^{NG} defines the unit cost of natural gas.

Maintenance cost (*MC*) is determined by the energy output from each device and the corresponding unitary maintenance cost (C^{maint}), as displayed in Eq. (8).

$$MC = \sum_i \sum_s \sum_h (E_{i,s,h}^{\text{pv/CHP}} + Q_{i,s,h}^{\text{hp/b-heat}} + Q_{i,s,h}^{\text{ec/ac-cool}} + Q_{i,s,h}^{\text{in-st}} + E_{i,s,h}^{\text{in-st}}) \times C_t^{\text{maint}} \quad \forall i, s, h \tag{8}$$

where $E^{\text{pv/CHP}}$ is electricity generated from PV panels and CHP, $Q^{\text{ec/ac-cool}}$ represents cooling energy supply by electric chiller or absorption chiller, $E^{\text{in-st}}$ and $Q^{\text{in-st}}$ are cooling and electricity in storage, respectively.

Grid cost (*GC*) can be derived from Eq. (9), depending on the electricity purchasing cost and the revenue generated by the surplus electricity sold to the grid.

$$GC = C_h^{\text{im}} \times \sum_i \sum_s \sum_h E_{i,s,h}^{\text{im}} - C_h^{\text{ex}} \times \sum_i \sum_s \sum_h E_{i,s,h}^{\text{ex}} \quad \forall i, s, h \tag{9}$$

where C^{im} and C^{ex} are unit price of grid electricity purchasing and tariff for electricity sold back to grid, respectively. E^{im} and E^{ex} represent the quantity of electricity purchased and sold.

Food income (*FI*) is determined by the sales income of food produced from the rooftop agriculture system and the agriculture system operational cost (Eq. (10)).

$$FI = \sum_{i}\sum_{k} A_i \times \varphi_{i,k}^{\text{agri}} \times (\text{income}_{i,k} - \text{opex}_{i,k}) \quad \forall i,k \tag{10}$$

where A_i is the rooftop area of each zone, $\text{income}_{i,k}$ represents the annual income per unit areas (m^2) for different rooftop agriculture options, and $\text{opex}_{i,k}$ indicates the operational cost of rooftop planting.

Eq. (11) defines the annualized carbon emissions (*ACE*) objective.

$$ACE = \psi_{\text{NG}} \times \sum_{i}\sum_{s}\sum_{h}\left(\frac{E_{i,s,h}^{\text{CHP}}}{\eta^{\text{CHP}}} + \frac{Q_{i,s,h}^{\text{b-heat}}}{\eta^{\text{b}}}\right) + \psi_{\text{grid}} \times \sum_{i}\sum_{s}\sum_{h} E_{i,s,h}^{\text{im}} + \sum_{i}\sum_{k} \psi_{k}^{\text{agri}} \times A_i \times \varphi_{i,k}^{\text{agri}} \tag{11}$$

where Ψ_{NG}, Ψ_{grid} and Ψ^{agri} are the embedded GHG emissions of natural gas, utility grid, and different rooftop agriculture options, respectively, where negative GHGs can be induced by rooftop agriculture options due to carbon sequestration.

Constraints. The formulated MILP optimization model is subject to following equality and inequality constraints.

Three energy balances have been introduced in the MILP model, i.e., electrical, heating and cooling balance. Eq. (12) constrains the heat balance, in which the energy saving potentials ($Q^{\text{h-roof}}$) of different rooftop agriculture options due to the rooftop thermal insulation effects are modelled as user-defined parameters, which can be calculated based on the methods given in previous studies (Li and Li, 2018; Nadal et al., 2017).

$$\begin{aligned}&(Q_{i,s,h}^{\text{h-dem}} - \sum_{k=1,2,3} \varphi_{i,k}^{\text{agri}} \times Q_{i,s,h,k}^{\text{h-roof}}) + \sum_{j} Q_{i,j,s,h}^{\text{hf}(i,j)} + Q_{i,s,h}^{\text{ac-heat}} = \\ &Q_{i,s,h}^{\text{re-heat}} + Q_{i,s,h}^{\text{hp}} + Q_{i,s,h}^{\text{b-heat}} + \sum_{j} Q_{j,i,s,h}^{\text{hf}(j,i)} \times (1 - \text{Lo}^{\text{h-pipe}}) \quad \forall i,j,s,h,j \neq i\end{aligned} \tag{12}$$

where $Q^{\text{h-dem}}$ represents heating demand of one zone. φ^{agri} is binary variable determining whether a rooftop agriculture option (k) is chosen. $Q^{\text{hf}(i,j)}$ is the heating energy flow from zone i to j via network, $Q^{\text{ac-heat}}$ is the heating consumed by absorption chiller, $Q^{\text{b-heat}}$ denotes the heating generated by boiler, $Q^{\text{re-heat}}$ is the heating recovered from CHP power generation, Q^{hp} represents the heating generated by heat pump, and $Lo^{h\text{-}pipe}$ is the heat loss coefficient of the heating network.

In the cool balance, the left-hand side of Eq. (13) includes cooling demand ($Q^{\text{c-dem}}$), potential cooling demand reduction ($Q^{\text{c-roof}}$) by implementing rooftop agriculture options (φ^{agri}), cooling charge (Q^{cha}) into a cooling storage, and the cooling energy flowing from zone i to j ($Q^{\text{cf}(i,j)}$). The right-hand side items are the cooling energy supply by electrical chillers ($Q^{\text{ec-cool}}$) and absorption chillers ($Q^{\text{ac-cool}}$), the cooling energy flowing from zone j to i ($Q^{\text{cf}(j,i)}$); and the cooling energy discharged (Q^{disc}) from the storage. A cooling loss rate ($Lo^{c\text{-}pipe}$) is applied to represent the cooling transfer loss.

$$
\begin{aligned}
&(\mathrm{Q}_{i,s,h}^{\text{c-dem}} - \sum_{k=1,2,3} \varphi_{i,k}^{\text{agri}} \times \mathrm{Q}_{i,s,h,k}^{\text{c-roof}}) + Q_{i,s,h}^{\text{cha}} + \sum_{j} Q_{i,j,s,h}^{\text{cf}(i,j)} = \\
&Q_{i,s,h}^{\text{ac-cool}} + Q_{i,s,h}^{\text{ec-cool}} + \sum_{j} Q_{j,i,s,h}^{\text{cf}(j,i)} \times (1 - \mathrm{Lo}^{\text{c-pipe}}) + Q_{i,s,h}^{\text{disc}} \quad \forall i,j,s,h, j \neq i
\end{aligned}
\tag{13}
$$

Eq. (14) defines the electrical balance. The energy consumptions by electrical chiller (E^{ec}), and heat pump (E^{hp}) together with electricity sold to the grid (E^{ex}) and battery charging ($E^{\text{st-in}}$) are equivalent to the sum of the electrical power discharged from battery storage ($E^{\text{st-out}}$), energy generation by PV panels (E^{pv}), on-site CHP (E^{CHP}) and electricity purchased from grid (E^{im}).

$$
E_{i,s,h}^{\text{ec}} + E_{i,s,h}^{\text{ex}} + E_{i,s,h}^{\text{hp}} + E_{i,s,h}^{\text{st-in}} + E_{i,s,h}^{\text{dem}} = E_{i,s,h}^{\text{st-out}} + E_{i,s,h}^{\text{pv}} + E_{i,s,h}^{\text{im}} + E_{i,s,h}^{\text{CHP}} \quad \forall i,s,h \tag{14}
$$

In addition to the above system integration-related constraints, other physical constraints for energy systems have been modelled and presented aa follows, including capacity limits (energy outputs constrained by the installed capacities), conversion constraints (cooling/heating/electricity energy conversion), storage constraints (constraints on both battery storage and thermal storage), operation constraints (on/off and ramp-up/down control), utility grid connections, and network constraints (logical constraints on energy network design and operation) (Jing et al., 2019c).

The energy conversions are derived in Eq. 15(a ~ e).

$$
Q_{i,s,h}^{\text{ec-cool}} = \eta^{\text{ec}} \times E_{i,s,h}^{\text{ec}} \quad \forall i,s,h \tag{15a}
$$

$$
Q_{i,s,h}^{\text{ac-cool}} = \eta^{\text{ac}} \times Q_{i,s,h}^{\text{ac-heat}} \quad \forall i,s,h \tag{15b}
$$

$$
Q_{i,s,h}^{\text{hp}} = \eta^{\text{hp}} \times E_{i,s,h}^{\text{hp}} \quad \forall i,s,h
$$

$$
Q_{i,s,h}^{\text{b-heat}} = \eta^{\text{b}} \times NG_{i,s,h}^{\text{b}} \quad \forall i,s,h \tag{15c}
$$

$$
E_{i,s,h}^{\text{CHP}} = \eta^{\text{CHP}} \times NG_{i,s,h}^{\text{CHP}} \quad \forall i,s,h \tag{15d}
$$

$$
Q_{i,s,h}^{\text{re-heat}} = \text{H-to-P} \times E_{i,s,h}^{\text{CHP}} \quad \forall i,s,h \tag{15e}
$$

The power output of solar PV panels is defined at Eq. 16.

$$
\eta_{s,h}^{\text{pv}} = P_1 \times [(\frac{SRI_{s,h}}{SRI_0})^{P_2} + P_3 \times (\frac{SRI_{s,h}}{SRI_0})][1 + P4 \times (\frac{T_{s,h}}{T_0})^{P_2} + P_5 \times \frac{AM_{s,h}}{AM_0}] \quad \forall s,h \tag{16a}
$$

$$
E_{i,s,h}^{\text{pv}} = \varphi_i^{\text{pv}} \times A_i \times \eta_{s,h}^{\text{pv}} \times SRI_{s,h} \quad \forall i,s,h \tag{16b}
$$

where the efficiency of PV panel η_{pv} is related to solar radiation index (*SRI*) in the unit of (W/m^2), the ambient temperature (*T*), and the air mass (*AM*), $SRI_0 = 1000$ W/m^2, $T_0 = 25$℃, $AM_0 = 1.5$, $P_1 = 0.2820$, $P_2 = 0.3967$, $P_3 = -0.4473$, $P_4 = -0.093$, $P_5 = 0.1601$; A_i is the available roof area.

To keep the linearity of the model, the efficiency of each energy supply device is assumed to be constant. Consequently, specific operation constraints are applied for the CHP avoiding low part-load operations and possible efficiency drop.

The minimum part load constraint is set at 30% of full capacity to avoid CHP operating at a low load range when the engine is on.

$$
E_{i,s,h}^{\text{CHP}} \leq \beta_{i,s,h}^{\text{CHP}} \times M_1 \quad \forall i,s,h \tag{17a}
$$

$$E_{i,s,h}^{\mathrm{CHP}} \geq (\beta_{i,s,h}^{\mathrm{CHP}} - 1) \times M_2 + 0.3 \times CAP_i^{\mathrm{CHP}} \quad \forall i,s,h \tag{17b}$$

where CAP^{CHP} is CHP installed capacity, and β^{CHP} is a binary variable for controlling the on/off status of CHP ($\beta^{\mathrm{CHP}} = 1$ is on). In addition, M_1 and M_2 are both big enough values to achieve the linear model.

To avoid frequently on/off of CHP, only one time on/off is allowed as derived in Eq. 18.

$$\sum_h \chi_{i,s,h}^{\mathrm{CHP}} \leq 1 \quad \forall i,s,h \tag{18a}$$

$$\chi_{i,s,h}^{\mathrm{CHP}} \geq \beta_{i,s,h}^{\mathrm{CHP}} - \beta_{i,s,h-1}^{\mathrm{CHP}} \quad \forall i,s,h \tag{18b}$$

$$\chi_{i,s,h}^{\mathrm{CHP}} \leq 1 - \beta_{i,s,h-1}^{\mathrm{CHP}} \quad \forall i,s,h \tag{18c}$$

$$\chi_{i,s,h}^{\mathrm{CHP}} \leq \beta_{i,s,h}^{\mathrm{CHP}} \quad \forall i,s,h \tag{18d}$$

where χ is a binary variable controlling the maximal frequency of switching on/off.

To avoid irrational fluctuation of CHP's power output mathematically, the power output fluctuation between last and this time-step cannot be larger than 50% of CHP's installed capacity.

$$E_{i,s,h}^{\mathrm{CHP}} - E_{i,s,h-1}^{\mathrm{CHP}} \leq 0.5 \times CAP_i^{\mathrm{CHP}} \quad \forall i,s,h \tag{19a}$$

$$E_{i,s,h-1}^{\mathrm{CHP}} - E_{i,s,h}^{\mathrm{CHP}} \leq 0.5 \times CAP_i^{\mathrm{CHP}} \quad \forall i,s,h \tag{19b}$$

In this study, both battery and cooling storage are available. Due to the similarity of storage devices' constraints, Eq. 20(a ~ e) takes the cooling storage as an illustrative example. The storage balance is constrained by Eq. 20a considering energy charge (η^{cha}), discharge (η^{disc}) and in-storage ($\eta^{\mathrm{in\text{-}st}}$) efficiency. The cooling in storage tank ($Q^{\mathrm{in\text{-}st}}$) at each time-step should less than or equal to the installed capacity of the storage tank (CAP^{st}). In the meantime, a binary variable (α) is introduced to avoid the cooling energy charging and discharging simultaneously.

$$Q_{i,s,h}^{\mathrm{in\text{-}st}} = \eta^{\mathrm{in\text{-}st}} \times Q_{i,s,h-1}^{\mathrm{in\text{-}st}} + \eta^{\mathrm{cha}} \times Q_{i,s,h}^{\mathrm{cha}} - Q_{i,s,h}^{\mathrm{disc}} / \eta^{\mathrm{disc}} \quad \forall i,s,h \tag{20a}$$

$$Q_{i,s,h}^{\mathrm{in\text{-}st}} \leq CAP_i^{\mathrm{st}} \quad \forall i,s,h \tag{20b}$$

$$Q_{i,s,h}^{\mathrm{cha}} \leq \alpha_{i,s,h}^{\mathrm{cha}} \times \overline{Q_{i,s,h}^{\mathrm{cha}}} \quad \forall i,s,h \tag{20c}$$

$$Q_{i,s,h}^{\mathrm{disc}} \leq \alpha_{i,s,h}^{\mathrm{disc}} \times \overline{Q_{i,s,h}^{\mathrm{disc}}} \quad \forall i,s,h \tag{20d}$$

$$\alpha_{i,s,h}^{\mathrm{disc}} + \alpha_{i,s,h}^{\mathrm{cha}} \leq 1 \quad \forall i,s,h \tag{20e}$$

The power exchange between utility grid and the integrated energy system is defined by:

$$0 \leq E_{i,s,h}^{\mathrm{ex}} \leq \delta_{i,s,h}^{\mathrm{ex}} \times \overline{E_{i,s,h}^{\mathrm{ex}}} \quad \forall i,s,h \tag{21a}$$

$$0 \leq E_{i,s,h}^{\mathrm{im}} \leq \delta_{i,s,h}^{\mathrm{im}} \times \overline{E_{i,s,h}^{\mathrm{im}}} \quad \forall i,s,h \tag{21b}$$

$$\delta_{i,s,h}^{\mathrm{ex}} + \delta_{i,s,h}^{\mathrm{im}} \leq 1 \tag{21c}$$

where δ^{ex} and δ^{im} are binary variables to control the power exchange and to avoid power export and import simultaneously.

This section describes network constraints, cooling and heating network constraints are similar from the modelling perspective. The heating energy can only transfer when two zones are connected via heating pipework as defined in Eq. 22a. Moreover, the connection between two zones should less than one time as derived in Eq. 22b.

$$\sum_j Q_{i,j,s,h}^{\mathrm{hf}(i,j)} \le \delta_{i,j}^{\mathrm{DH}} \times \overline{Q_{i,j,s,h}^{\mathrm{hf}(i,j)}} \quad \forall i,j,s,h,j \ne i \tag{22a}$$

$$\delta_{i,j}^{\mathrm{DH}} + \delta_{j,i}^{\mathrm{DH}} \le 1 \quad \forall i,j \ne i \tag{22b}$$

where δ^{DH} is a binary variable indicating whether the connection exists or not among zones (1 is connected, 0 is not).

Similarly, cooling transfer can only happen if cooling pipework exists among zones as derived in Eq. 23.

$$\sum_j Q_{i,j,s,h}^{\mathrm{cf}(i,j)} \le \delta_{i,j}^{\mathrm{DC}} \times \overline{Q_{i,j,s,h}^{\mathrm{cf}(i,j)}} \quad \forall i,j,s,h,j \ne i \tag{23a}$$

$$\delta_{i,j}^{\mathrm{DC}} + \delta_{j,i}^{\mathrm{DC}} \le 1 \quad \forall i,j \ne i \tag{23b}$$

At each time step, each zone (i) cannot simultaneously receive and transfer energy to others (j) as constrained by Eq. 24.

$$\sum_j Q_{i,j,s,h}^{\mathrm{cf}(i,j)} \le \gamma_{i,s,h}^{\mathrm{DC}} \times \overline{Q_{i,j,s,h}^{\mathrm{cf}(i,j)}} \quad \forall i,j,s,h,j \ne i \tag{24a}$$

$$\sum_j Q_{j,i,s,h}^{\mathrm{cf}(j,i)} \le (1-\ \gamma_{i,s,h}^{\mathrm{DC}}) \times \overline{Q_{j,i,s,h}^{\mathrm{cf}(j,i)}} \quad \forall i,j,s,h,j \ne i \tag{24b}$$

$$\sum_j Q_{i,j,s,h}^{\mathrm{hf}(i,j)} \le \gamma_{i,s,h}^{\mathrm{DH}} \times \overline{Q_{i,j,s,h}^{\mathrm{hf}(i,j)}} \quad \forall i,j,s,h,j \ne i \tag{24c}$$

$$\sum_j Q_{j,i,s,h}^{\mathrm{hf}(j,i)} \le (1-\ \gamma_{i,s,h}^{\mathrm{DH}}) \times \overline{Q_{j,i,s,h}^{\mathrm{hf}(j,i)}} \quad \forall i,j,s,h,j \ne i \tag{24d}$$

where γ^{DC} is a binary variable controlling the status of transfer or receive.

The optimization model was developed in GAMS 25.0.3 and solved by the CPLEX solver on 8*12 – Core Xeon X5675 clusters with 48GB RAM. The optimality gap is set to 1%, and all other settings remain at default values.

Supplemental Reference

Abdalla, M., Wattenbach, M., Smith, P., Ambus, P., Jones, M., and Williams, M. (2009). Application of the DNDC model to predict emissions of N2O from Irish agriculture. Geoderma *151*, 327-337.

Babu, Y.J., Li, C., Frolking, S., Nayak, D.R., and Adhya, T.K. (2006). Field validation of DNDC model for methane and nitrous oxide emissions from rice-based production systems of india. Nutr Cycl Agroecosyst *74*, 157-174.

Beheydt, D., Boeckx, P., Sleutel, S., Li, C.S., and Van Cleemput, O. (2007). Validation of DNDC for 22 long-term N2O

field emission measurements. Atmos Environ *41*, 6196-6211.

Benis, K., Reinhart, C., and Ferrão, P. (2017). Development of a simulation-based decision support workflow for the implementation of Building-Integrated Agriculture (BIA) in urban contexts. Journal of Cleaner Production *147*, 589-602.

Brown, L., Syed, B., Jarvis, S.C., Sneath, R.W., Phillips, V.R., Goulding, K.W.T., and Li, C. (2002). Development and application of a mechanistic model to estimate emission of nitrous oxide from UK agriculture. Atmos Environ *36*, 917-928.

Butterbach-Bahl, K., Kesik, M., Miehle, P., Papen, H., and Li, C. (2004). Quantifying the regional source strength of N-trace gases across agricultural and forest ecosystems with process based models. Plant Soil *260*, 311-329.

Butterbach-Bahl, K., Stange, F., Papen, H., and Li, C.S. (2001). Regional inventory of nitric oxide and nitrous oxide emissions for forest soils of southeast Germany using the biogeochemical model PnET-N-DNDC. J Geophys Res-Atmos *106*, 34155-34166.

Cai, Z.C., Sawamoto, T., Li, C.S., Kang, G.D., Boonjawat, J., Mosier, A., Wassmann, R., and Tsuruta, H. (2003). Field validation of the DNDC model for greenhouse gas emissions in East Asian cropping systems. Glob Biogeochem Cycle *17*, 10.

CMDC (2018). China meteorological data sharing service system (http://data.cma.cn).

Gilhespy, S.L., Anthony, S., Cardenas, L., Chadwick, D., del Prado, A., Li, C., Misselbrook, T., Rees, R.M., Salas, W., Sanz-Cobena, A., *et al.* (2014). First 20 years of DNDC (DeNitrification DeComposition): Model evolution. Ecological Modelling *292*, 51-62.

Grant, B., Smith, W.N., Desjardins, R., Lemke, R., and Li, C. (2004). Estimated N2O and CO2 emissions as influenced by agricultural practices in Canada. Paper presented at: 12th International Soil Conservation Organization Conference (ISCO) (Beijing, PEOPLES R CHINA: Kluwer Academic Publ).

Guo, M., Li, C., Bell, J.N.B., and Murphy, R.J. (2012). Influence of Agro-Ecosystem Modeling Approach on the Greenhouse Gas Profiles of Wheat-Derived Biopolymer Products. Environmental Science & Technology *46*, 320-330.

Guo, M., Li, C., Facciotto, G., Bergante, S., Bhatia, R., Comolli, R., Ferré, C., and Murphy, R. (2015). Bioethanol from poplar clone Imola: an environmentally viable alternative to fossil fuel? Biotechnol Biofuels *8*, 1-21.

Jing, R., Kuriyan, K., Kong, Q., Zhang, Z., Shah, N., Li, N., and Zhao, Y. (2019a). Exploring the impact space of different technologies using a portfolio constraint based approach for multi-objective optimization of integrated urban energy systems. Renewable and Sustainable Energy Reviews *113*, 109249.

Jing, R., Wang, M., Zhang, Z., Liu, J., Liang, H., Meng, C., Shah, N., Li, N., and Zhao, Y. (2019b). Comparative study of posteriori decision-making methods when designing building integrated energy systems with multi-objectives. Energy and Buildings *194*, 123-139.

Jing, R., Wang, M., Zhang, Z., Wang, X., Li, N., Shah, N., and Zhao, Y. (2019c). Distributed or centralized? Designing district-level urban energy systems by a hierarchical approach considering demand uncertainties. Applied Energy *252*, 113424.

Jing, R., Zhu, X., Zhu, Z., Wang, W., Meng, C., Shah, N., Li, N., and Zhao, Y. (2018). A multi-objective optimization and multi-criteria evaluation integrated framework for distributed energy system optimal planning. Energy Conversion and

Management *166*, 445-462.

Li, C., Frolking, S., and Frolking, T.A. (1992). A model of nitrous oxide evolution from soil driven by rainfall events: 1. Model structure and sensitivity. Journal of Geophysical Research: Atmospheres *97*, 9759-9776.

Li, C., Salas, W., Zhang, R., Krauter, C., Rotz, A., and Mitloehner, F. (2012). Manure-DNDC: a biogeochemical process model for quantifying greenhouse gas and ammonia emissions from livestock manure systems. Nutr Cycl Agroecosyst *93*, 163-200.

Li, H., and Li, X. (2018). Benchmarking energy performance for cooling in large commercial buildings. Energy and Buildings *176*, 179-193.

Liang, L., Ridoutt, B.G., Wu, W., Lal, R., Wang, L., Wang, Y., Li, C., and Zhao, G. (2019). A multi-indicator assessment of peri-urban agricultural production in Beijing, China. Ecological Indicators *97*, 350-362.

Mavromatidis, G., Orehounig, K., and Carmeliet, J. (2018). Uncertainty and global sensitivity analysis for the optimal design of distributed energy systems. Applied Energy *214*, 219-238.

Nadal, A., Llorach-Massana, P., Cuerva, E., López-Capel, E., Montero, J.I., Josa, A., Rieradevall, J., and Royapoor, M. (2017). Building-integrated rooftop greenhouses: An energy and environmental assessment in the mediterranean context. Applied Energy *187*, 338-351.

Perera, A.T.D., Nik, V.M., Chen, D., Scartezzini, J.-L., and Hong, T. (2020). Quantifying the impacts of climate change and extreme climate events on energy systems. Nature Energy.

Smith, W.N., Desjardins, R.L., Grant, B., Li, C., Lemke, R., Rochette, P., Corre, M.D., and Pennock, D. (2002). Testing the DNDC model using N2O emissions at two experimental sites in Canada. Can J Soil Sci *82*, 365-374.

Unternährer, J., Moret, S., Joost, S., and Maréchal, F. (2017). Spatial clustering for district heating integration in urban energy systems: Application to geothermal energy. Applied Energy *190*, 749-763.

Wang, Y.P., Meyer, C.P., Galbally, I.E., and Smith, C.J. (1997). Comparisons of field measurements of carbon dioxide and nitrous oxide fluxes with model simulations for a legume pasture in southeast Australia. J Geophys Res-Atmos *102*, 28013-28024.

Yue, X., Pye, S., DeCarolis, J., Li, F.G.N., Rogan, F., and Gallachóir, B.Ó. (2018). A review of approaches to uncertainty assessment in energy system optimization models. Energy Strategy Reviews *21*, 204-217.

Supplemental Figures

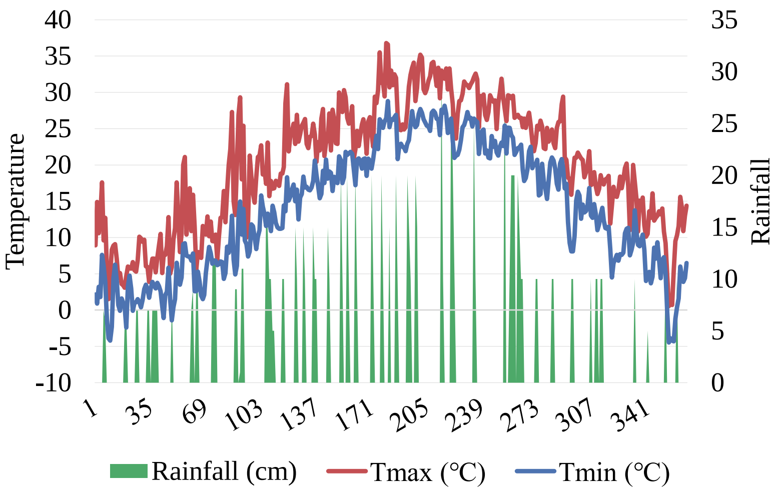

Fig S1 Daily maximum and minimum temperature and rainfall conditions for DNDC simulations, Related to Table 2. Tmax – maximum temperature (℃), Tmin – minimum temperature (℃), rainfall (cm).

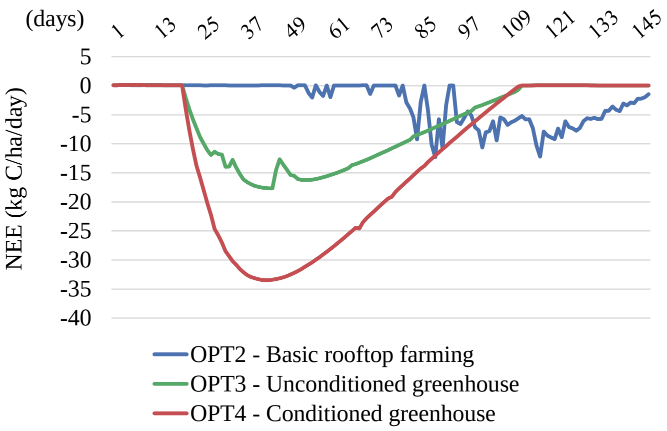

Fig S2 DNDC simulated daily NEE fluxes for one crop cycle (approximately 150 days) for different rooftop agriculture options, Related to Table 2.

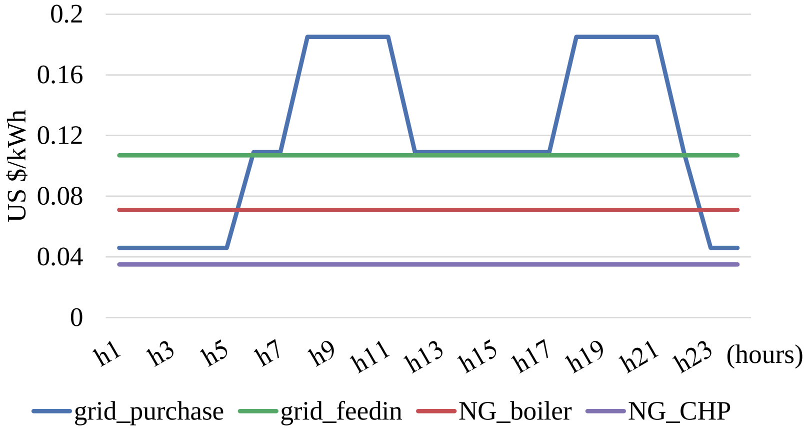

Fig S3 Different categories of time-of-use energy prices, Related to Figure 4, 5, 6, and 7. Tariffs includes the peak/non-peak electricity purchase tariff, constant electricity feed-in tariff, and different prices for CHP or boiler natural gas (NG) consumption.

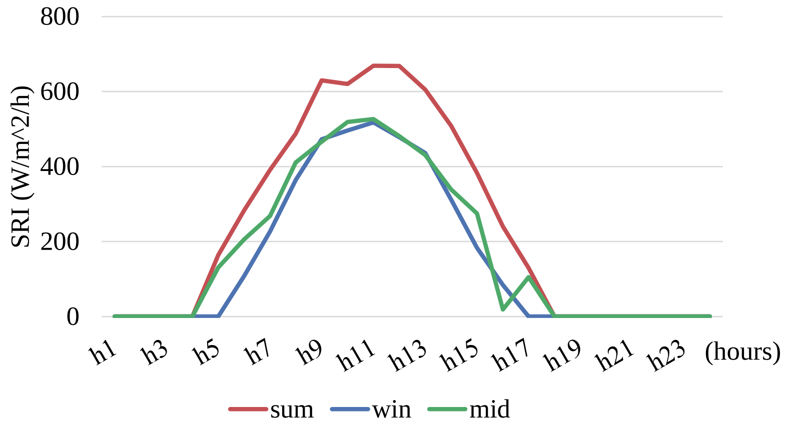

Fig S4 Solar radiation index for different seasons (sum – summer, win – winter, mid – transition), Related to Figure 4, 5, 6, and 7.

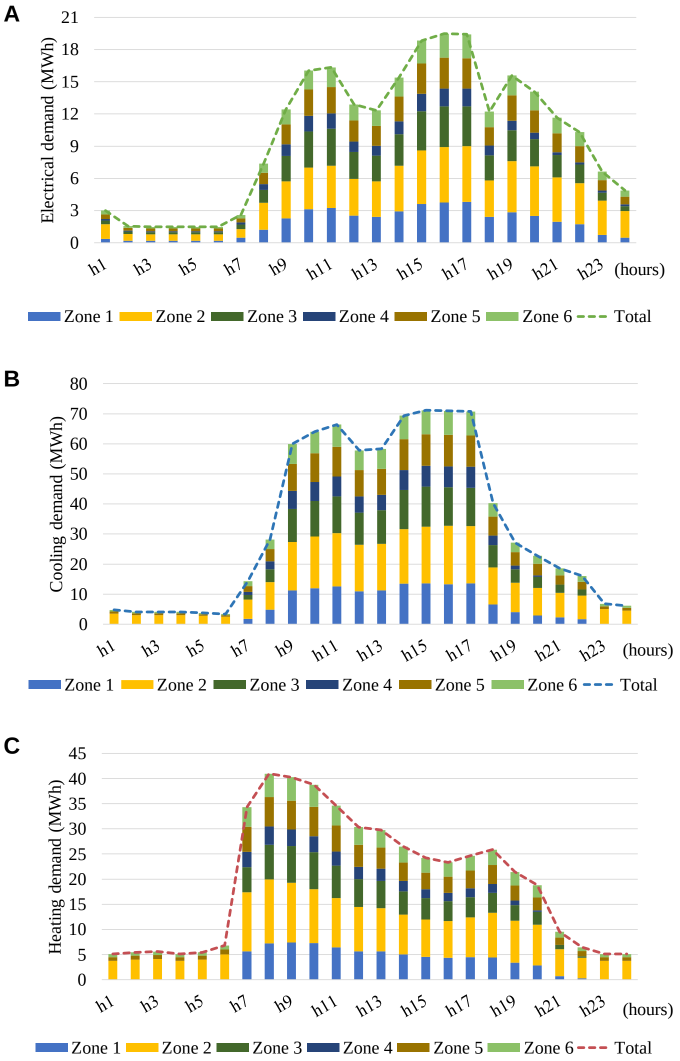

Fig S5 Hourly energy demand breakdown by zones, Related to Figure 4, 5, 6, and 7. Assuming the electricity demand for winter, summer, and transition seasons are similar as shown in Fig. S5A. The typical day cooling demand is shown in Fig. S5B, which only happens in summer. The corresponding heating demand in winter is shown in Fig. S5C.

Supplemental Tables

Table S1 Plant parameters for DNDC simulations with varying management strategies, Related to Table 2

Plant parameter	Value			Notes
	OPT2 – Basic roof farming	OPT3 – Unconditioned greenhouse	OPT4 – Conditioned greenhouse	
Maximum biomass yield	210	505	950	kg C/ha/yr
Biomass fraction	0.36/0.22/0.22/0.2	0.36/0.22/0.22/0.2	0.36/0.22/0.22/0.2	fruit/leaf/stem/root
C/N ratio	26/26/26/45	26/26/26/45	26/26/26/45	fruit/leaf/stem/root
Total N demand	205	525	929	kg N/ha/yr
Thermal degree days (TDD)	1400	1400	1400	°C
Water requirement	1300	3120	5822	kg water/kg dry matter
N fixation	1	1	1	Plant N/N taken from soil
Optimum temperature	25	25	25	°C
Fertilization	Precision	Precision	Precision	Auto applied if needed
Irrigation	Auto	Auto	Auto	Irrigation index 0.9
CO_2 concentration	350	350	500	ppm
Controlled temperature if available	NA	NA	25	°C

Table S2 Cost coefficients for three rooftop agriculture options, Related to Figure 4, 5, 6, and 7 (Benis et al., 2017; Liang et al., 2019)

	Basic roof farming (OPT2)	Unconditioned greenhouse (OPT3)	Conditioned greenhouse (OPT4)
Capital cost ($\$/m^2$)	15	30	45
Fertilizer ($\$/m^2/y$)	0.06	0.60	1.10
Water ($\$/m^2/y$)	0.42	0.31	0.48
Energy ($\$/m^2/y$)	0.20	0.40	2.00
Substrate ($\$/m^2/y$)	0	0.23	0.50
Labor ($\$/m^2/y$)	2.85	4.50	8.00
Pesticides ($\$/m^2/y$)	0	0.83	1.20
Operation total ($\$/m^2/y$)	3.53	6.87	13.28

Table S3 A list of parameters applied in the optimization model, Related to Figure 4, 5, 6, and 7

Parameters	Definitions	Value
$C_{\mathrm{CHP}}^{\mathrm{CAP}}$	Unit capital cost of CHP [$/kW]	1,000
$C_{\mathrm{b}}^{\mathrm{CAP}}$	Unit capital cost of boiler [$/kW]	60
$C_{\mathrm{ec}}^{\mathrm{CAP}}$	Unit capital cost of electric chiller [$/kW]	120
$C_{\mathrm{ac}}^{\mathrm{CAP}}$	Unit capital cost of absorption chiller [$/kW]	170
$C_{\mathrm{hp}}^{\mathrm{CAP}}$	Unit capital cost of heat pump [$/kW]	140
$C_{\mathrm{pv}}^{\mathrm{CAP}}$	Unit capital cost of PV panel [$/kW]	650
$C_{\mathrm{pipe}}^{\mathrm{CAP}}$	Unit capital cost of heating and cooling network [$/m]	200
$C_{\mathrm{b\text{-}st}}^{\mathrm{CAP}}$	Unit capital cost of battery storage [$/kWh]	2,000
$C_{\mathrm{c\text{-}st}}^{\mathrm{CAP}}$	Unit capital cost of cooling storage tank [$/kWh]	35
C_{k}^{CAP}	Unit capital cost of k rooftop agriculture option [$\$/m^2$]	See Table S3
$DX_{i,j}$	Distance between zones	See Fig. 2A
η^{CHP}	Efficiency of CHP (ele)	0.4
H-to-P	Heat-to-power rate of CHP	0.75
η^{b}	Efficiency of boiler	0.85
η^{ec}	Efficiency of electric chiller	4
η^{ac}	Efficiency of absorption chiller	1.2
η^{hp}	Efficiency of heat pump	2.5
η^{pv}	Efficiency of PV panel	0.14
$\eta^{\mathrm{in\text{-}st}}$	Efficient of cooling storage self-discharge	0.9
$\eta^{\mathrm{cha/disc}}$	Efficient of cooling storage charge/discharge	0.9
$C_{h}^{\mathrm{CHP\text{-}NG}}$	Unit cost of natural gas for CHP [$/kWh]	See Fig. S3
$C_{h}^{\mathrm{b\text{-}NG}}$	Unit cost of natural gas for boiler [$/kWh]	See Fig. S3
$C_{\mathrm{CHP}}^{\mathrm{maint}}$	Maintenance cost of CHP [$/kWh]	0.003

$C_{\mathrm{b}}^{\mathrm{maint}}$	Maintenance cost of boiler [$/kWh]	0.0003
$C_{\mathrm{ec}}^{\mathrm{maint}}$	Maintenance cost of electric chiller [$/kWh]	0.001
$C_{\mathrm{ac}}^{\mathrm{maint}}$	Maintenance cost of absorption chiller [$/kWh]	0.001
$C_{\mathrm{hp}}^{\mathrm{maint}}$	Maintenance cost of heat pump [$/kWh]	0.001
$C_{\mathrm{pv}}^{\mathrm{maint}}$	Maintenance cost of PV panel [$/kWh]	0.003
$C_{\mathrm{b\text{-}st}}^{\mathrm{maint}}$	Maintenance cost of battery storage [$/kWh]	0.003
$C_{\mathrm{c\text{-}st}}^{\mathrm{maint}}$	Maintenance cost of cooling storage tank [$/kWh]	0.0003
CRF	Capital recovery factor for 15, 25, 30 years	0.103, 0.085, 0.073
C_{h}^{im}	unit price of grid electricity purchasing at hour h [$/kWh]	See Fig. S3
C_{h}^{ex}	tariff for electricity sold back to grid at hour h [$/kWh]	See Fig. S3
Ψ_{grid}	Emission factor of the grid electricity [kg/kWh]	0.55
Ψ_{NG}	Emission factor of natural gas power generation [kg/kWh]	0.18
ψ_{k}^{agri}	Emission factor of k rooftop agriculture option	See Table 2
A_i	Available roof area in i zones [m^2]	900~2,500
$\mathrm{income}_{i,k}$	Income for k rooftop agriculture option in zone i [$/m^2/year]	3.71, 8.87, 16.26
$\mathrm{Q}_{i,s,h}^{\mathrm{h\text{-}dem}}$	Heating demand in zone i at season s and hour h	See Fig. S5
$\mathrm{Q}_{i,s,h}^{\mathrm{c\text{-}dem}}$	Cooling demand in zone i at season s and hour h	See Fig. S5
$\mathrm{Q}_{i,s,h,k}^{\mathrm{h\text{-}roof}}$	Heating demand saved in zone i by k rooftop agriculture option	4%, 5%, 6% of the original demand
$\mathrm{Q}_{i,s,h,k}^{\mathrm{c\text{-}roof}}$	Cooling demand saved in zone i by k rooftop agriculture options	4%, 5%, 6% of the original demand
$Lo^{\mathrm{c\text{-}pipe}}$	Cooling network thermal loss rate	6%
$Lo^{\mathrm{h\text{-}pipe}}$	Heating network thermal loss rate	5%
$SRI_{s,h}$	Solar Radiation index at season s and hour h	See Fig. S4
